# Face yourself: The social neuroscience of mirror gazing

**DOI:** 10.3389/fpsyg.2022.949211

**Published:** 2022-11-10

**Authors:** Antonella Tramacere

**Affiliations:** ^1^Department of Philosophy and Communication Studies, University of Bologna, Bologna, Italy; ^2^Department of Linguistic and Cultural Evolution, Max Planck Institute for the Science of Human History (MPI-SHH), Jena, Germany

**Keywords:** mirror gazing, body image, body positivity, self-image, social neuroscience, social psychology, self-perception

## Abstract

In philosophical and psychological accounts alike, it has been claimed that mirror gazing is like looking at ourselves *as* others. Social neuroscience and social psychology offer support for this view by showing that we use similar brain and cognitive mechanisms during perception of both others’ and our own face. I analyse these premises to investigate the factors affecting the perception of one’s own mirror image. I analyse mechanisms and processes involved in face perception, mimicry, and emotion recognition, and defend the following argument: because perception of others’ face is affected by our feelings toward them, it is likely that feelings toward ourselves affect our responses to the mirror image. One implication is that negative self-feelings can affect mirror gazing instantiating a vicious cycle where the negative emotional response reflects a previously acquired attitude toward oneself. I conclude by discussing implications of this view for psychology and social studies.

## Feelings in the mirror

When we perceive others’ people in ecological situations, we may do a number of different things: we may mimic their facial expressions and resonate with their emotions, we may empathize or sympathize with them, or show appreciation or depreciation to them. Also, the way we feel toward others affects the way we perceive them and behavioral reactions towards them. For example, if we appreciate someone for their biography or personality, we will likely respond with positive emotions and prosocial behavior to their face (van Baaren et al., 2009; Franzen et al., 2018); on the contrary, if we do not like the other person, we may more probably lack to show sympathy and emotional connection to them.

What happens when we perceive our own self? What do we do, for example, when we look at our own face?

We cannot see our own face directly, but we can see it reflected in a mirror. Because of its autoscopic function, the mirror has fascinated human beings for centuries (Pendergrast, 2009). Through mirrors, we can perceive the visible aspects of our own face and body as others can see them and acquire an externalized perspective on ourselves. The mirror image is an objectified representation of ourselves and allow seeing us as through the gaze of an another.

Because mirror images embody an externalized perspective on the self, the ability to recognize oneself in the mirror has been considered the mark of a self-concept, namely a well-integrated, flexible, and conscious representation of the self as a being in the world (Gallup, 1977). The mirror test (Gallup, 1970, 1979), where experimenters place a dot on the forehead of the subject to see whether they try to touch or remove the dot, has been developed to inquire into animals and children ability to recognize themselves as themselves in the mirror, and to determine whether and when they become self-conscious.

In the last decades, this view has been challenged, and individuals who are able to recognize their body in the mirror are no longer considered to necessarily possess a self-concept (Heyes, 1994; Suddendorf and Butler, 2013). Further, variability has been found across human groups in mirror self-recognition, with different cultural groups showing different responses to the mirror test as consequence of their different practices with the mirror (Broesch et al., 2011). Not all cultures use the mirror for self-identification, and individuals with no experience with the mirror may interpret their mirror image differently (Rochat, 2009). For example, the Buryats of Eastern Mongolia conceive of mirrors as instruments that implement luminosity in the house and that enrich the display of precious objects; therefore, it is questionable whether individuals in these cultures have familiarized themselves with the mirror as a self-identifying tool (Humphrey, 2007).

Notwithstanding, in many societies the mirror is used as a tool to observe visible aspects of one’s own body. In addition, the spread of self-directed pictures (aka selfie) in many countries across the globe suggests that, at least in these countries, most individuals recognize themselves as themselves in mirroring surfaces because of a process of socio-cultural learning (Rettberg, 2014). Consequently, for many individuals who are socialized with the mirror as a self-identifying tool, it makes sense to ask what factors affect the experience of mirror gazing (i.e., looking at ourselves in a mirroring surface).

Social psychology and neuroscience show that mechanisms of self-face perception are similar to mechanisms of others’ face perception (Decety and Sommerville, 2003; Uddin et al., 2005; Bretas et al., 2021). This suggests that we perceive both ourselves and others by using common neurocognitive processes (brain and psychological mechanisms; Gallese, 2003). Neurocognitive findings about self and others’ face perception are compatible with the idea that in the mirror we perceive the otherness of ourselves by adopting an external perspective on our own face; when we observe our own face in the mirror, we see it as it were the face of another. I will call this externalized perspective on one’s own face “the social coding of mirror gazing.” My contentious is that in the social coding of mirror gazing lay the keystone of the mirror as a tool of self-knowledge to inquire into how acquired feelings toward ourselves affect visual self-perception.

Findings in social psychology show that when we perceive others, our affective attitudes toward them modulate our responses to their face: consciously or unconsciously appreciating others affects whether, and to what extent, we respond with positive or negative emotions and corresponding facial mimicry (McIntosh, 2006; Bourgeois and Hess, 2008; van Baaren et al., 2009). Based on this premise, and on the similarity between the mechanisms of self and others’ face perception, I argue that the perceptual processing of the mirror image is very likely influenced by the affective attitudes toward oneself. In other words, the way we feel toward ourselves affects the perception of ourselves in the mirror, and our behavioural and emotional responses to the mirror image.

The way we represent and evaluate our mirror image has important societal implications. It is widely claimed that a positive attitude toward oneself is connected to social wellbeing. For example, several social media and cultural movements advocate the importance of accepting and appreciating one’s own self to acquire a positive *body image*, where the latter is typically defined as the perceptual and conceptual representation of, and the affective attitude toward one’s own body (Sastre, 2014). Also, educators and psychologists in many different countries around the world acknowledge that *body positivity* is not a negligible feature of our psychological life, and it is pivotal for social well-being (Tylka and Wood-Barcalow, 2015).

Much work has been done to identify nature and origins of body image disturbance and eating disorders (Soh et al., 2006), to analyse possible psychological causes of these phenomena and social power dynamics involved in their emergence across groups and individuals (Sepúveda et al., 2002). However, to my knowledge less philosophical attention has been devoted to *whether* and *how* individuals’ responses to the mirror image are affected by self-related feelings. One consequence of this is that the processes through which our feelings may have an impact on how we perceive and evaluate ourselves in the mirror are insufficiently investigated. My analysis aims to fill this gap.

In this paper, I investigate how affective attitudes may affect mirror self-perception through interpretations of the dynamics of top-down processes (from attitudes to perceptual responses) and the interplay between emotion and cognition. I take literally the hypothesis of a social coding of the mirror gazing, hence of an objectifying perspective on our mirror image, to studying phenomena of visual self-representation through social psychology. Consequently, perceiving ourselves as others becomes more than simply a metaphor describing our mirror experience, but rather an occasion to inquire into how what we think about ourselves affects self-perception.

The plan of the article is the following: in Section 2, I discuss results from social neuroscience showing that similar neurocognitive processes are activated during both self and others’ face observation. In Section 3, I describe studies showing that others’ face observation is affected by non-perceptual factors, such as affective attitudes toward others. In Section 4, I argue that mirror gazing is affected by the affective attitudes toward us. In Section 5 I discuss objections to this argument, and then conclude.

## The brain in face perception

*Similar* brain mechanisms are activated during the observation of one’s own face and of others’ faces, where the similarity regards common brain location and neural circuitry. By discussing evidence from social neuroscience, I will show that neural regions with similar physiological properties activate during both self-face observation *and* other face observation (common coding) and that the regions of the brain that code for both self and others’ faces are part of common brain networks (common neural circuitry).

**Figure 1:**
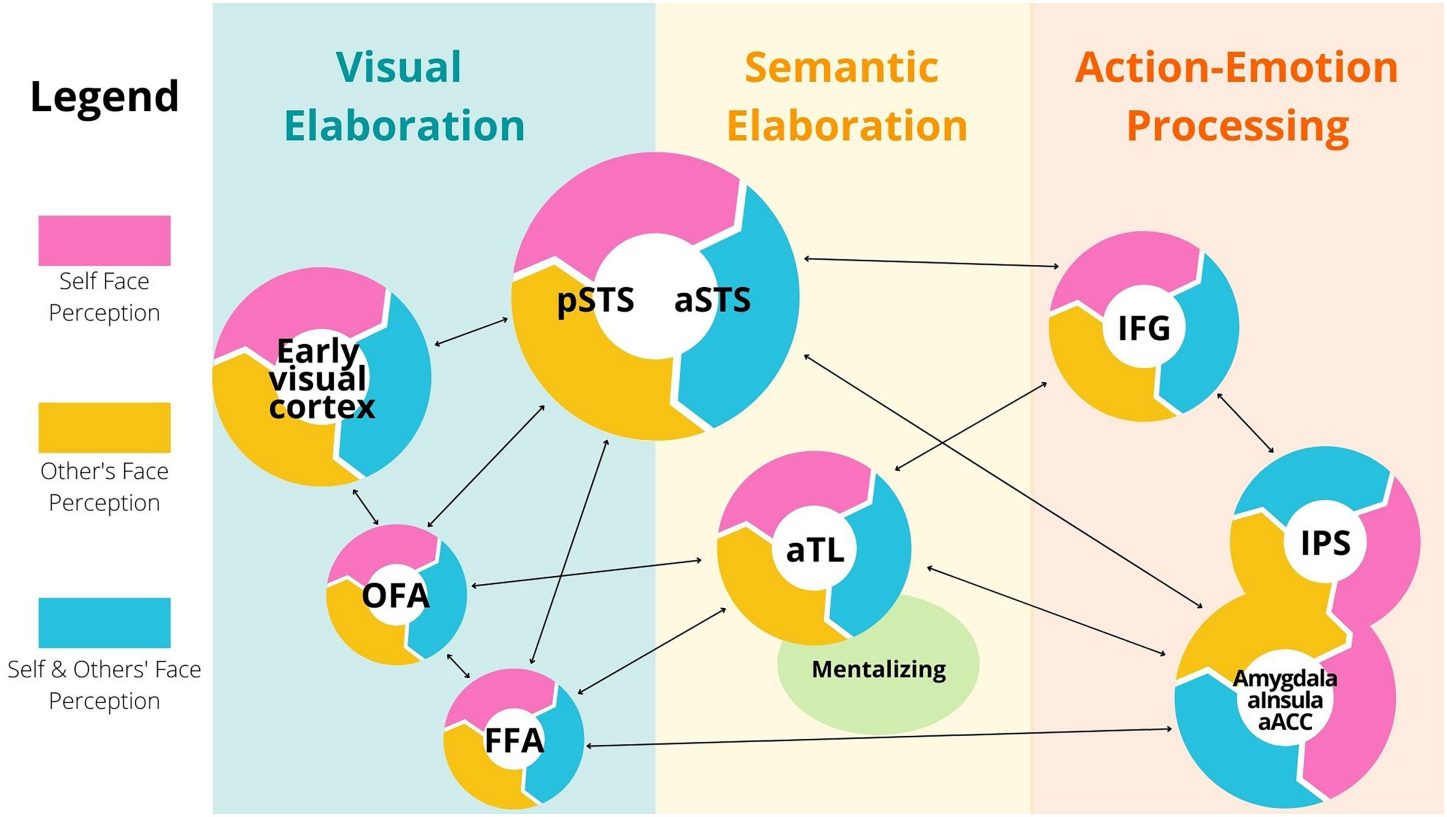
Simplified model of face selective areas. Different brain regions activate in a hierarchical and parallel fashion during perception of one’s own face and during perception of others’ faces. These regions are categorized by visual (light blue), semantic (light orange), action and emotion-centered elaboration (red). In this network, each region possesses both self-face perception areas (pink), others’ face perception areas (yellow) and areas that activate in both conditions (blue). These distinctions are meant to reflect probabilistic activations during task performances, and not domain-specific functions of the brain. Abbreviations: OFA: Occipital Face Area, FFA: Face Fusiform Area, pSTS and aSTS: posterior and anterior Superior Temporal Sulcus respectively, aTL: anterior Temporal Lobule, IFG: Inferior Frontal Gyrus, IPS: Inferior Parietal Sulcus, aInsula: anterior Insula, ACC: Anterior Cingulate Cortex.

I based the following discussion on the Haxby et al. (2000) model about the face processing network, its extension in the Duchaine and Yovel (2015) model and additional relevant studies. Through this body of research, we learned that specific areas of the primary visual cortex, plus regions of the occipital cortex, such as the Occipital Face Area (OFA), are active during the early visual elaboration of faces^1^ (Figure 1, left blue panel). OFA is thought to code invariant structural features of faces, and to be involved in attribution of identity (Ambrus et al., 2017a,b). Additional important regions of the face processing network are the Fusiform Face Areas (FFA), and the anterior and posterior regions of the Superior Temporal Sulcus, respectively aSTS and pSTS [Figure 1, left (blue) and central (yellow) panels]. While the FFA is involved in holistic coding of faces, the anterior and posterior regions of STS multimodally code face expressions, through fine-grained processing of head, lip, and eye motion (McCarthy et al., 1997; Hoffman and Haxby, 2000). STS seems to be robustly involved in emotion recognition of perceived faces (Hoffman and Haxby, 2000; Engell and Haxby, 2007; Wang et al., 2016). In contrast, the function of FFA is still under debated, as it is yet unclear whether this area is involved in identity attribution, emotion recognition or both^2^ (Bernstein and Yovel, 2015).

Crucial for my argument here is that specific populations of neurons in the above mentioned face selective regions (OFA, FFA, aSTS and pSTS) are active during both self-face and others’ face perception (Kircher et al., 2000; Platek et al., 2006; Figure 1, light blue sectors). There is no doubt that, in these brain areas, neurons also activate either during self-face or during others’ face observation (Figure 1, yellow and pink sectors). In fact, although the areas typically active during perception of both self and other familiar faces are adjacent *and* partially overlapping, it is possible to experimentally disambiguate between them. Further, some studies found a right hemisphere dominance for self-face perception compared to others’ face perception [even though other studies found equivalent bilateral activations for both self and others discrimination, probably reflecting differences in task context; see Platek et al. (2006) for a discussion of the latter point].

These findings are not surprising. We need to distinguish between different faces, and perceiving differences between self and others’ faces also requires activating different brain and cognitive mechanisms. However, I do not think that these differences undermine the claim that brain regions coding for others’ faces are similar to regions coding for self-face perception, because neurons active during both self and others’ face observation are located in the same area, and they also share equivalent neurophysiological properties.

Sensorimotor and somatosensory neurons in frontal and parietal areas, as well as in the limbic system are also active during both self- and others’ face perception (Uddin et al., 2005; Bretas et al., 2021). To support the idea that brain sensorimotor and somatosensory mechanisms for others’ face perception are similar to those active for self-face perception, I will mainly discuss evidence regarding the mirror neuron system (MNS).

The MNS is a neural network including frontal and parietal areas [the frontoparietal circuits, comprising inferior frontal gyrus (IFG) and the inferior parietal sulcus (IPS)], but also limbic areas [anterior insula, anterior cingulate cortex (ACC) and the amygdala; Uddin et al., 2005; see Figure 1, red panel]. This wide cortical–subcortical network is called “mirror” because it contains many neurons with the key property of being active during both execution and perception of similar behaviour, including facial expressions (Ferrari et al., 2003, 2017). During face processing, wide neural populations in the temporal, limbic and frontoparietal circuits are active and code for fine-grained aspects of face actions and emotions, both in a social (allocentric) and individual (egocentric) condition (Uddin et al., 2005).

How do the mirror properties of frontoparietal and limbic regions of the brain support the claim that both self and others’ face perception are coded by similar neurocognitive mechanisms? As I said, the MNS is a distributed network of neurons with mirroring properties, which are active both during observation and execution of self and others’ face. Consider a smile. While you smile, a wide-spanning network of sensorimotor and somatosensory neurons activate and are associated with the time-course of your smile, coding both kinematics and valence information (Manjula et al., 2015). When you observe someone else smiling, a part of this network is also active and constitute an action-perception matching system for social cognition (Caruana et al., 2017). This basic matching mechanism underlie *perception* of disgust in self and others (Wicker et al., 2003), as well as pain (Timmers et al., 2018), laughter and joy (Caruana et al., 2017). Thus, perceiving others’ facial expressions activates motor and somatosensory areas involved in the execution of the same facial behavior (Schilbach et al., 2008; Likowski et al., 2012).

Crucial for my argument is that sensorimotor and somatosensory neurons with mirror properties are also active while you observe your own face in the mirror. Studies have shown that the key areas of the frontoparietal network are activated during self-face movements and others’ face perception, as well as during self-face perception (Decety and Sommerville, 2003). While watching their own face in the mirror, individuals activate portions of the frontoparietal MNS that are activated during others’ faces perception (Platek et al., 2004, 2006; Uddin et al., 2005). Further, single neurons in the superior parietal cortex are active both during self-body observation in the mirror, during observations of others’ body and during tactile perception of self-body (Bretas et al., 2021), suggesting that a similar coding mechanism might be present for face perception. Thus, beyond visual areas in the occipital and temporal cortices, also sensorimotor and somatosensory regions of the frontoparietal cortex activate during both self-face observation and others’ face observation [Figure 1, right red panel].

The MNS is not the only associative circuit active both during self-face observation and others face observation. Selective areas of the anterior temporal lobule (aTL) and the mentalizing system, comprising dorsal prefrontal cortex, temporoparietal junction and anterior paracingulate cortex, are also active while we look at faces, in both an allocentric and egocentric perspective (Feinberg, 2001; Haxby et al., 2004; Morita et al., 2008; Platek et al., 2008; see Figure 1, yellow panel). During social cognitive tasks, regions in the MNS, the anterior temporal cortex, and the mentalizing systems work in conjunction. Cortical and subcortical neural nodes of the MNS are often involved in lower-order social cognitive mechanisms, such as identifying kinematic and affective aspects of observed behavior (Keysers et al., 2014; Urgesi et al., 2014; Carrillo et al., 2019). In contrast, the anterior temporal lobe and the nodes of the mentalizing system are thought to be involved in the semantic interpretation of others’ goals, emotions and beliefs (Wong and Gallate, 2012; Hyatt et al., 2015).

The neurophysiological properties of the occipital and the temporal areas of the brain, the MNS and the mentalizing system active during various aspects of face perception support the view that both self and others’ face observation are coded by common brain circuits. Again, as in the case of occipital and temporal cortices, also for the MNS and the mentalizing system, I talk of commonality and not of sameness, because neurons coding for one’s own face and others’ face are not identical nor 100% overlapping. I contend that the listed commonalities between brain mechanisms for self and others’ face are sufficient for the generalization of functions from social cognition to mirror gazing.

## Feelings in face-to-face interactions

In this section, I will analyze a series of studies supporting the view that the affective attitudes toward a person affects the perception of their face. Affective attitudes can be defined as feeling toward a person (or an object) and may include conscious and unconscious emotions for that person, and explicit evaluations about them such as appreciation for their biography or personality. As such, affective attitudes can be associated to many types of mental states, be propositional and non-propositional, conceptual and non-conceptual one^3^. Based on this definition, I will discuss studies that analyze how feelings toward a person affect perception of their face, even when those studies do not mention or define affective attitudes as I do here.

Affective attitudes toward others bias the perceptual process of their facial expressions, and this bias involves perceptual, emotional, and sensorimotor components. For example, previously acquired information about others affects neural processes in the occipitotemporal regions of the brain while perceiving their face (Abdel Rahman, 2011; Abdel Rahman and Sommer, 2012; Wieser et al., 2014). Knowing that someone is a rapist reduces activity in the visual STS during perception of their face, compared to when we think that they are kind people (Galli et al., 2006). These studies are yet inconclusive regarding the exact visual correlates of these changes. It is also unclear whether social information is processed by agents in rational or prerational terms, thus making it uncertain what level of mentalizing is required for the modulation of the perceptual process. All we know is that the differences in sensory regions of the observers correlate with the positive and negative recognition bias that they manifest during others’ facial expressions.

When facial expressions are ambiguous, emotion recognition is biased along with priming of emotional descriptions, such as happy or sad (Halberstadt et al., 2009; Zhao et al., 2017). Biographic information with high emotional value about unknown individuals affects not only our eventual appreciation of them, but also the recognition of their facial expressions (Suess et al., 2015). In other words, affective knowledge about a person affects emotional responses to them and biases the recognition of their facial expressions toward the valence of the previously acquired information.

During the perception of others’ faces, we seem to spontaneously retrieve information about the perceived person (Todorov et al., 2007). Interestingly, this information modulates our behavioral responses: individuals show a positive bias while perceiving facial expressions previously associated with positive personality features, by recognizing faster and more accurately happy faces than negative ones. At the same time, a negative bias is found with faces previously associated with negative personality features, because subjects are usually more accurate in categorizing negative expressions such as anger and sadness (Bijlstra et al., 2014; Albohn and Adams Jr., 2016). Interestingly, different behavioral responses during others’ face perception are reflected in different patterns of brain activations (Abdel Rahman, 2011; Abdel Rahman and Sommer, 2012; Luo et al., 2016).

Individuals are more likely to manifest emotional contagion with people they like and feel emotionally connected to (Krebs, 1975). Emotional contagion occurs when an observer responds to others’ emotional behavior with the same emotional expression (Zillman and Cantor, 1977; McIntosh, 2006). On the contrary, during the perception of strangers’ faces, or of faces of people with whom there is no emotional connection, individuals show less emotional contagion (van Baaren et al., 2009). These responses have been detected quite robustly, and interestingly they often are conveyed bodily through changes in facial expressions, such as facial mimicry.

Facial mimicry occurs when individuals automatically react with same covert or overt facial movements to the facial behavior of others. Consider again a smile: smiling requires the joint activation of a series of facial muscles, controlling among other things the movements of the lip corner and of the ocular parts, such as the Orbicularis oculi and the Zygomaticus mayor (Manjula et al., 2015). When you perceive someone smiling, your Orbicularis oculi and Zygomaticus mayor muscles will also be activated. The story, as often is told in biology and psychology, is not so simple and linearly determined. Facial mimicry is modulated by a variety of factors (Bourgeois and Hess, 2008; Kraaijenvanger et al., 2017), and there is a bidirectional relationship between facial mimicry and social knowledge: Individuals mimicking more in response to others’ facial expressions are normally rated as more likable and are more likely to trigger sympathy in the social partner (Duffy and Chartrand, 2015). In other words, responding with more facial muscles’ activation during face-to-face interactions with others is likely going to make individuals nicer; at the same time, previously acquired sympathy or positive attitudes toward a person modulate the phenomenon of mimicry while perceiving their face (McIntosh, 2006; Bourgeois and Hess, 2008; Likowski et al., 2008; Kraaijenvanger et al., 2017).

When we observe the facial behavior of strangers or individuals we do not particularly like, we generally show less facial mimicry (Lakin and Chartrand, 2003). Consider one interesting and quite old study (McHugo et al., 1985), which inquired into individuals’ facial reactions to a Reagan’s speech. The study found through electro magnetoencephalography that observers who did not support the U.S. President showed less activity in the cheek, and more corrugator brow activity than his supporters when viewing him smiling, suggesting that the perception of the smile of an enemy inhibits our mimicry response, and rather can bias us toward the expression of anger.

A series of studies confirms this trend, showing that people sympathy for the actor was correlating with the degree of facial mimicry showed (Chartrand and van Baaren, 2009; Duffy and Chartrand, 2015). The general pattern found was that more sympathy correlate with higher activation of cheek and mouth muscles involved in smiling and happy facial expression when the observed person was smiling and showing happiness. Similarly, during observed negative emotions in others, individuals were reacting with higher mimicry involving sad facial expressions. In contrast, when individuals have low sympathy for a person, they show less facial mimicry during perception of positive emotions, and increased tendency expression of negative emotions.

Interestingly, quite robust evidence suggests that the MNS is causally involved in phenomena of facial mimicry and emotional contagion (Hogeveen et al., 2015; Kraaijenvanger et al., 2017; Paz et al., 2022). This has been shown in the last decades through studies that have inquired simultaneously into the activity of the brain with more than one neuroscientific tool (Likowski et al., 2012). The simultaneous use of different neuroscience techniques with different direction of bias is often employed to disambiguate controversial results about causal questions (Tramacere, 2021). Although questions on the exact and functional role of the MNS remain, the claim that the MNS is causally involved in facial mimicry and related perception of others’ emotion is relatively well established.

Note that evidence showing MNs activation during mimicry and emotional contagion does not imply that no other perception-motor neurons are active or important for explaining these phenomena. Further, the involvement of the MNS does say nothing on the functional model used to explain their effect and it is in principle compatible with different hypotheses (such as the simulation, direct perception and predictive coding hypothesis; Michael, 2011). The robust activation of the MNS during facial mimicry and emotional contagion episodes only says that the mechanisms of action-perception matching served by this system is important in explaining social phenomena based on face-to-face interactions (Tramacere and Ferrari, 2016).

## Feeling toward the mirror image

The perception of our own face in the mirror may be affected by similar types of non-perceptual factors which modulate others’ face perception in ecological situations. Specifically, the affective attitude toward ourselves can affect facial perceptual processing, as well as behavioural and psychological responses to our own mirror image. Therefore, affective self-attitudes have an impact on behavioral and psychological responses during self-face observation, and eventually what we know about the social brain can be instructive to inquire into the experience of mirror gazing.

During mirror gazing, individuals may activate regions of the social brain that convey responses in line with the internalized affective attitude toward themselves. The neurophysiological and circuitry properties of face perception network make plausible that as certain affective attitudes toward others bias us toward corresponding behavioural and emotional responses during their face perception, affective attitudes toward ourselves can bias behavioural and emotional responses to our own face in the mirror.

If my argument is correct, a negative way of representing oneself could produce negative emotions and corresponding facial expressions during mirror self-recognition. For example, an aversive self-image could (perhaps unconsciously) bias individuals’ facial expressions toward certain emotions (sadness, disgust, and anger), and corresponding covert facial mimicry. Further, individuals with aversive self-image could show a higher activation of facial muscles normally activated during sadness, anger, or disgust, while an opposite bias could be found in subjects with positively connoted self-image. Note that while in the case of social cognitive responses, we could talk of emotional contagion and facial mimicry with the observed others, in the case of self-perception these concepts can only be used in a metaphorical way.

In the case of mirror gazing, negative behavioral and psychological responses toward oneself could be considered a *sui generis* form of emotional contagion, where the emotion that the subject resonates to relates to their own emotional attitude toward themselves. The emotional response to the mirror image may also trigger automatic and fast mimicry facial responses, so that the subject also covertly and unconsciously activates facial muscles that correspond to negative emotional responses, such as contempt, disgust, anger, or sadness. In other words, a self-sustaining vicious circle could be instantiated during mirror gazing, involving various forms of negative responses toward oneself.

As far as I am aware, no studies tested this specific hypothesis. However, various psychological studies are compatible with and provide broad support for my claim. For example, an extensive range of studies have showed that body concerns and self-esteem are bidirectionally related (Feingold, 1992; O’Dea, 2012; Felisberti, 2014). In one study (Oikawa et al., 2012) people reported low self-appreciation and low self-esteem when their image was compared with individuals who were rated as more attractive, suggesting that the affective attitudes toward oneself is not fix across time, but dynamical and influenced by contextual factors. In this study, self-face appreciation was associated with activation of the reward system, while negative self-evaluation modulated areas of the face perception network, supporting the view that a positive self-image produces positive feelings.

One study (Jauk et al., 2017) inquired into whether personality disorders can affect self-face evaluation, and the findings suggest that this might be the case. The authors showed that, compared to typical subjects, subjects with high scores of narcissism have greater activation in areas of the brain which are typically correlated with expectancy violation and negative emotion. Another study (Potthoff and Schienle, 2021) performed with eye-tracking show that subjects with low self-esteem look longer at their own face, possibly reflecting a higher critical gaze on oneself.

Further studies have found correlations between affective self-knowledge and psychological and behavioral responses during perception of one’s own face and body. One study tested emotional responses to distorted self-face perception, where subjects rated altered images of their own face as more embarrassing than the altered image of others. Interestingly, while recognition of self-face correlated with the activity of the action MNS, changes in embarrassment were co-varying with activity of both the MNS and the mentalizing system (Morita et al., 2008). Another recent study (Maister et al., 2021) inquired into the pictorial visual representation of individuals and compared it with various self-construal index, and showed that the valence of individuals self-representation correlated with self-attributed visual features.

A shortcoming of these studies is that the causal direction of interaction is not inquired about; therefore, other causal factors could produce self-directed emotions with, e.g., negative valence, and the emotional attitude toward oneself could be a consequence, and not a cause of those results. However, the validity of my argument does not require that no other factors can affect self-face perception and associated behavioural and psychological responses, nor it requires that self-perceived physical and psychological characteristics are univocally, rather than bidirectionally, related. I will engage with objections to my argument in the next section.

## Objections

There could be objections to the argument that feelings toward oneself affect the perception of one’s own face and corresponding behavioral and psychological responses. I will consider two main objections: (1) causal effect from non-perceptual processes (such as affective attitudes) to mirror gazing are unlikely, because the emotional ways we represent objects cannot exert influence on perceptual responses; (2) even if (somehow) responses to others’ face could be influenced by affective attitudes toward others, this process cannot generalize to perception of oneself.

I will address these objections in turn, beginning with (1). Someone could object that although some scholars claim that non-perceptual content affects perception (Stokes, 2013), this claim is still controversial and is not supported by conclusive arguments nor evidence. Furthermore, the objection would continue, none of the studies that I have discussed here conclusively show that non-perceptual content, such as attitudes, have a direct causal and semantic influence on perceptual responses, such as visual perception and object representation. Therefore, according to this objection, the conclusion that affective attitudes influence responses during observation of one’ own face in the mirror is wrong or at least unsupported.

I think this objection is out of target. It is true that, in my analysis, I consider modulations from non-perceptual processes, such as affective attitudes, to perceptual mechanisms. However, I am not claiming that this modulation regards low-level, basic visual properties of the observed object, such as the invariant aspects of face identity perception. I am not analyzing the impact of higher-level content (beliefs, intentions, and desires) on low-level visual coding of face. My argument is tangential to cognitive penetration debate, which regards whether cognition affects perceptual processes, with perception being narrowly defined as a purely sensory, non-interpretative process, and cognition being defined as elaboration in propositional and conceptual terms.

My focus is rather mostly on higher-order processes of perception, where the multimodal sensory coding of a percept (i.e., faces) overlaps and intermingles with motor and affective coding. I have thus embraced here a rich conception of perception (Burnston, 2017, in press) and inquired into how these higher-level features of perception (seeing as) are connected to behavioral and psychological responses of an individual (e.g., emotional responses and facial mimicry). If you agree that perceptual processes do not necessarily occur in encapsulated and domain-specific areas of the brain which are insensitive to processes in other areas and domains, and if you allow perceptual responses to involve and recruit emotional, affective, and motor responses, the objection that attitudes cannot affect perception will lose force.

Recall previously discussed evidence. We have seen that during observations of others’ face, brain processes in the occipitotemporal cortices are modulated by the ways we represent others. We have seen however that it is unclear what are the functional correlates of those changes. Similarly, it is likely that during the observation of self-face, patterns of changes in the facial perceptual stream in the occipitotemporal lobe correlate with the valence of attitude toward oneself, but I do not think we can make any reasonable prediction about what these changes are in the subjects’ eyes.

Further, during observation of others’ face, brain changes in somatosensory and sensorimotor areas predict patterns of facial mimicry and emotional responses to others’ people face, and these responses are modulated by affective attitudes toward others. Based on the arguments I offered in previous sections, it is reasonable that having aversive self-image produce changes in the face processing network, and that these changes bias the activation of negative expressions, such as sadness. Note however that even though this prediction will be verified, and that we can find a correlation between negative self-image and sadness during mirror perception, I do not think that we can be sure that the subject of this experience is feeling sad. It is possible that a subject showing brain activation, bodily markers and facial mimicry responses normally correlated with sadness also feels sad, but this is not obvious. Like in the case of others’ face perception, during perception of one’s own face the emotional response to oneself could remain unconscious, thus making the mental state attribution to the subject arduous. Although we cannot identify the exact mental correlates of subjects’ behavioral and emotional responses during mirror gazing, we can base our analyses on the probabilistic relations between facial expression and associated emotions to narrow the space of inferences about subjects’ experiences with the mirror.

Let us see objection (2). One could claim that even if affective attitudes toward others may influence responses of the observer during others’ face perception, this does not allow generalizing this premises to self-face observation. On this objection, although the brain mechanisms for self and others’ face observations are similar, (i) their activation says little on the similarity between self and others’ face representation at the psychological level. Further, (ii) similar brain mechanisms for self and others’ face perception do not ensure that they are affected by similar psychological mechanisms, such as, feeling toward oneself. Affective attitudes toward oneself and others may be instantiated by different mechanisms, and we do not know whether they can modulate subjects’ responses to one’s own face, as they modulate responses to others’ face.

Regarding (i), note that while I have examined the similarities between brain mechanisms of self- and other’s face perception in an analytical way, a broad range of studies already provide support that self and others’ face perception share many similar aspects at the psychological levels; these studies suggest that we recognize others’ faces through psychological mechanisms that are similar to those while discriminating our own faces (Rochat, 2009; Rochat et al., 2012; Porciello et al., 2018). To contradict this claim, one should demonstrate that the perception of our own face has distinctive psychological features, and that these features prevent our own face perception to be modulated by emotions and feelings about ourselves. I honestly do not see any evidence pointing in this direction, and already existing studies support the conclusion that this is not the case [see for example Oikawa et al. (2012)].

The objection (ii) puts doubt that responding with, say, sadness to one’s own face during self-face observation is caused by generalized negative feelings toward oneself, rather than by alternative causes. The objection could add that it is possible individuals show dissociations between fast emotional/mimicry responses on the one hand, and explicit, reportable emotional attitudes toward oneself on the other hand. That is, if we observe bodily markers, brain activations and facial mimicry patterns that are normally correlated with sadness during mirror gazing, not only we cannot conclude that the subject is feeling sad, but we cannot even say that this response is caused by an aversive self-image. Sadness or other negatively connoted emotional responses to one’s own face could be caused by other psychological or non-psychological mechanisms, and not necessarily by aversive self-affective attitudes.

I agree that multiple causes can be responsible for aversive responses during mirror gazing. Further, I acknowledge the complexity and possible dissociation between automatic emotional responses and more reflective evaluative representation of oneself. I do not think however that this complexity speaks against the validity (and heuristic utility) of my argument. Several methods could be employed to make sure that the sad response of the subject is elicited by self-representation during mirror gazing, for example by priming subjects’ responses to others’ faces with valence information about oneself [one study in this direction is again Oikawa et al. (2012)].

While many methods can be employed for narrowing down the inferences that self-related affective attitude can bias responses to one’s own face, it is not the purpose of this paper to propose exactly which experimental methods can settle the debate. My interest here is only to provide good enough reasons for possible neurocognitive mechanisms that can explain whether and how attitudes toward oneself affect mirror gazing. This explanation can do further justice to psychological data that we already possess, and that can possibly describe social and cultural phenomena involving affective self-representation and perception of oneself in the mirror.

## Conclusion

For decades cognitive neuroscience has told us that we use parts of the brain involved in performing actions and emotions to perceive and understand actions and emotions of others. We know others through the neurocognitive structures that we use for moving, sensing, and feeling in the world (Cacioppo, 2006); but we also sense, feel, and get to know ourselves through the neurocognitive structures that we have acquired during affective and communicative interactions with others. Because we are not able to directly perceive our face through vision and the first knowledge that we acquired about faces derives from facial interactions with others, the reciprocity of self-other perception is especially relevant for self-face observation on mirroring devices.

In this paper, I have inquired about self-face perception through the lenses of social psychology and neuroscience. Analysing mirror gazing through social neuroscience does not aim at reducing the phenomenon of face self-perception to the activation of parts of the brain active during the visual perception of our own face. I instead considered neural and behavioral evidence as an occasion to enrich our understanding of the experience of mirror gazing and stimulate new thinking in the philosophy of mind and experimental psychology.

A social neuroscience approach to mirror gazing is centered on addressing what happens while we see our own face in mirroring devices, what responses we show in front of the mirror image, and whether these responses may say something about the way we represent ourselves. I have discussed studies supporting the view that similar neurocognitive mechanisms, in respect to both brain location and neural circuitry, are active for both self-face and others’ face perception. Specific activation in key visual areas, the action and emotion MNS and the mentalizing system suggest that during self-face observation, the neurocognitive mechanisms involved in the perceptual, action, and emotion coding of others’ face are modulated by non-perceptual variables, such as affective attitudes toward oneself. The reviewed studies suggest that self-affective attitudes could affect whether we respond with positive or negative emotions to oneself, and with a corresponding facial mimicry, and these responses could be mediated by the face processing areas, the action and emotion MNSs and the mentalizing system.

If the way we feel toward ourselves can produce a series of negative or positive emotional responses to the mirror image, they may trigger a vicious circle which involves various forms of antipathy and depreciation toward oneself. Since these responses are likely to be automatic and fast, this negative circle could be difficult to break. Therefore, the hypotheses I formulated imply that face-to-face interactions are relevant to the appreciation, and understanding of ourselves, and that the interactions with others are relevant to delineate the phenomenological experience with oneself. Mirror gazing would then necessarily be included in the boundary of social interactions, and how the gaze of others affects the perception and understanding of oneself.

I am convinced that the arguments proposed in this article can be informative for psychological and phenomenological research, by providing a heuristic parallel between social and self-related cognitive processes, which can explain important societal phenomena and pave the way to novel experimental predictions to be explored in future research. If my interpretations are correct, my arguments of a bias from affective attitude to self-face perception can provide an important basis for making sense of the rich experience of mirror gazing in many contemporary cultures.

According to some psychological studies (e.g., Perugi et al., 1997), individuals who show negative attitudes toward one’s own face or body are not necessarily considered ugly or unpleasant by others. In some cases, the perceptual distortion during self-observation can be so prominent to produce psychological disorders, such as dysmorphophobia, namely a psychological condition characterized by the phobia of being ugly and by the pathological use of mirrors, which can produce significant discomfort in the individuals that are affected by it (Veale and Riley, 2001). Because often no significant correlations have been found between objective bodily features (as evaluated by other individuals) and body-image, the hypothesis of a role of affective self-attitude in self-perception can be relevant to explain such phenomena. I contend that these hypotheses can provide an interesting basis for analysing the phenomenology of mirror gazing in individuals of different age and developmental history, and of the mirror as a tool for self-knowledge.

## Data availability statement

The original contributions presented in the study are included in the article, further inquiries can be directed to the corresponding author.

## Author contributions

The author confirms being the sole contributor of this work and has approved it for publication.

## Conflict of interest

The author declares that the research was conducted in the absence of any commercial or financial relationships that could be construed as a potential conflict of interest.

## Publisher’s note

All claims expressed in this article are solely those of the authors and do not necessarily represent those of their affiliated organizations, or those of the publisher, the editors and the reviewers. Any product that may be evaluated in this article, or claim that may be made by its manufacturer, is not guaranteed or endorsed by the publisher.

## Footnotes

^1^Following Haxby et al. (2000) models, the Occipital Face Area is considered the gateway to the face processing network. However, there is evidence that multiple pathways convey face representations into the network (Dalrymple et al., 2011; Yang et al., 2016).

^2^Note that there is ongoing discussion on whether one common or two separate routes exist for face identity and face emotion processing. The majority of neuroimaging studies address both face identity and face expression functions, plus data are mixed. However, a detailed discussion of the ongoing controversies on the function of the various visual areas responding to faces is beyond the scope of the article.

^3^I employ the philosophical concept of affective attitude to capture the entire spectrum of mental states that can be defined as feeling toward, hence states that regard the emotional salience of perceived objects and bias the perceptual process toward certain emotional modes. Employing psychological constructs such as "social knowledge" (e.g., knowledge about others) would instead force me to narrowly characterize the mental variables affecting perception of oneself in terms of conceptual content, while I want to remain open about the types of states that may influence the process of face perception. Because the way we feel toward someone or ourselves can be conveyed by both conceptual and non-conceptual content, I prefer to use the concept of affective attitudes in such a pluralistic way.
